# Maternal and infant risk factors and risk indicators associated with early childhood caries in South Africa: a systematic review

**DOI:** 10.1186/s12903-022-02218-x

**Published:** 2022-05-18

**Authors:** Faheema Kimmie-Dhansay, Robert Barrie, Tina Roberts, Sudeshni Naidoo

**Affiliations:** 1 Division of Research and Postgraduate Studies, Faculty of Dentistry, University of the Western Cape, Cape Town, South Africa; 2 Department of Community Oral Health, Faculty of Dentistry, University of the Western Cape, Cape Town, South Africa; 3 Department of Oral Biology and Dental Genetics, Faculty of Dentistry, University of the Western Cape, Cape Town, South Africa

**Keywords:** Risk, South Africa, Early childhood caries, Socio-economic status, Educational level, Family situation, Maternal characteristics

## Abstract

**Objectives:**

To evaluate the risk factors and risk indicators associated with early childhood caries in South Africa.

**Design:**

A systematic review of aetiology was performed. From 1366 papers found, 23 studies met the eligibility criteria and were included. All study designs were included. Healthy children under six who live in South Africa were eligible for the study. The study was registered with PROSPERO, registration number CRD42020216455.

**Data extraction:**

Eligible studies were selected, and data extracted independently by two reviewers. Published data on socio-economic status, dietary factors, oral hygiene knowledge and practices, breastfeeding and bottle-feeding practices, oral bacterial flora and other risk indicators were collected. Two authors appraised the studies independently using the Joanna Briggs Critical Appraisal tools.

**Data analysis:**

Heterogeneity was assessed using the I^2^ statistics, and due to heterogeneity, extracted data were mostly presented narratively.

**Results:**

Meta-analysis was performed using random-effects models and concluded that parents of children who had a tertiary education had a 1.77 [1.22–2.57] odds of experiencing dental caries compared to the children of parents with a secondary education. However, the unclear risk of bias of most included cross-sectional studies precluded definitive conclusions.

**Conclusions:**

More high-quality cohort studies need to be performed to evaluate actual risk factors for ECC in a South African setting. Parental/caregiver oral educational knowledge needs promoting before the emergence of their children’s teeth. The social determinants of health need to be incorporated in future studies, and suitable targeted interventions need to be developed and implemented to mitigate early childhood caries in South Africa.

**Supplementary Information:**

The online version contains supplementary material available at 10.1186/s12903-022-02218-x.

## Introduction

Early childhood caries (ECC) is a, “common disorder characterised by the presence of one or more decayed (non-cavitated or cavitated lesions), missing (due to caries), or filled tooth surface of primary teeth in children 71 months or younger. Severe early childhood caries (S-ECC) is either any smooth tooth surface decay in children under 3 years or one or more cavitated, missing (due to caries) or filled smooth tooth surface in the primary maxillary anterior teeth in children between the ages of 3 and 5 years”, according to the American Academy of Pediatric Dentistry [1].

Globally, maternal and infant risk factors have impacted the presence and severity of ECC. Socio-economic status, breastfeeding frequency, maternal depression, Vitamin D deficiency, toothbrushing frequency, oral hygiene knowledge, rural status, and toothbrushing frequency have been associated with the ECC. To determine the associations, cross-sectional study designs were primarily employed in South Africa (SA) as it was quick and cost-effective. However, these studies failed to answer the question of aetiology. Despite this knowledge, ECC remains a challenge, especially in South Africa, with a national prevalence of 50.6% [2]. A systematic review on the prevalence and severity of ECC in South Africa is about to be published and shows that ECC prevalence is high and increasing over time. There have been systematic reviews evaluating the risk factors associated with ECC [3–5]. However, only three studies from South Africa were included [6–8] and evaluated *Streptococcus Mutans*, sucrose intake and breast and bottle-feeding practices. Thus only a few risk factors were considered for a South African setting. To inform policymakers, the risk factors that affect ECC in South Africa need to be assessed to produce interventions explicitly targeted for South African children.

Due to the systematic harms of the apartheid regime, South Africa is one of the unequal societies in the world, with a Gini index of 0.65 in 2015 [9]. A Gini index of 0 indicates an equal society and a coefficient of 1 indicates complete inequality. Even though Apartheid was abolished almost 30 years ago, the lasting impact it has had on the population can be seen in the healthcare system, where the lower socio-economic and disadvantaged groups have higher levels of non-communicable and infectious diseases. According to Statistics South Africa, the vast majority, which forms the lower 60% of the population, receives their income from social grants other than the labour market [9].

Although South Africa has the highest total wealth in Africa and holds $552 billion in wealth, less than 3% of the country’s total expenditure is consumed by 20% of the South African population [10]. Furthermore, the wealthiest 20% consume 65% of the South African economy [10]. More impoverished communities often live in temporary housing structures without running water, electricity in gangster-riddled, drug-infested living environments. Furthermore, there is a major disjoint between medical and dental services between rural and urban areas and public and private healthcare systems.

South Africa spends about 8.25% of its Gross Domestic Product on health (about US$525.96 per capita). Of the total spending of R1.79 trillion spent in 2018/2019, 12% went to health, of which 4% went to public health (R72 billion), and 7% went to hospitals (R123 billion) [11]. Thus, most doctors opt to work for the private sector, leaving most of the under-funded public sector with limited access to health care. Access to health care is thus limited to those that can afford it, while the rest of the population must depend on the public healthcare system [12].

One of the biggest challenges in managing ECC in South Africa is the poor access to dental treatment. There is approximately a 1: 6000 dentist to person population ratio in the country. There is a free primary health care approach to basic dental care for the South African population which provides basic services such as oral examinations, radiographs, dental extractions and restorations and antibiotic prescriptions for adults and children [13]. However, access to general anaesthesia is rarely performed at the primary level of care. This implies that children under 6 are not all afforded the opportunity to treatment under general anaesthesia, and thus there is a long waiting list for extractions under GA [14]. It is thus imperative that ECC be prevented as to relieve the burden on our already overwhelmed healthcare system.

In South Africa, the maternal and infant risk factors and indicators must be studied and incorporated into mainstream health care as part of NCD prevention strategies. A risk factor is associated with temporal change, i.e., with longitudinal studies. A risk indicator is associated with cross-sectional studies. Longitudinal studies are rare due to their costs and the length of time to detect the outcome. This review hopes to elicit targeted interventions aimed at South African children to mitigate ECC. The present systematic review aimed to determine the maternal and infant risk factors and risk indicators associated with early childhood caries in a South African setting.

## Materials and methods

This systematic review is the first to evaluate the risk factors associated with ECC in SA. This study was conducted following the Meta-Analysis of Observational Studies in Epidemiology (MOOSE) guidelines. The protocol is registered with the International Prospective Register of Systematic Reviews (PROSPERO)—CRD42020216455, and the proposal was published [15]. The initial search produced 1366 articles, of which 193 were duplicates. The first assessment was performed by title and abstract, with irrelevant articles discarded at this stage. This process reduced the number of articles for full-text review to 48, of which 23 were considered for inclusion. The papers consisted of 19 cross-sectional and four cohort studies.

### Focused question

The research question was determined using the population of children younger than 6 years of age; exposure is the risk factor or risk indicators associated with the outcome, in this case, caries prevalence.

### The eligibility criteria

There were no limitations to language or year of publication. Studies were considered eligible for inclusion if participants met the following criteria: (a) Performed in South Africa (b) systemically healthy children,  < 6 years old; HIV is a confounder and thus was excluded from the study (c) assessed for dental caries using a validated tool such as ICDAS, dmft, dmfs and (d) recorded risk factors. Studies without risk factors were not included. All observational studies published in English were included in this review (cross-sectional and cohort studies). Unpublished manuscripts, conference abstracts and all other grey literature were excluded. Studies were not included in this review if the outcome of interest was not measured or reported.

### Outcome measures

Primary outcome measures were risk factors or risk indicators and dental caries, determined using a validated tool.

### Search methods for identification of studies

Further to an initial scoping exercise with a librarian, searches were conducted without restriction on language or year of publication in Scopus, Embase (Academic Search Complete, Cinahl, Dentistry and oral sciences), Science Direct, PubMed. The search in Pubmed used the following keywords and MeSH terms: a. “South Africa” b. children OR “perinatal” OR paediatric OR pediatric OR neonatal OR infant c. “risk factor*” OR risk or factor* d. “early childhood caries” OR caries OR decay OR dmft OR dental OR oral OR PUFA in the order a + b + c + d. References were imported into Rayyan to delete duplications and include or exclude studies based on eligibility [16]. There were no filters or limits in the search strategy. Reference lists of included articles were also checked for suitable studies to be included in the review.

### Screening and selection

All studies were screened by two trained reviewers (FKD and TR) by evaluating their titles and abstracts, independently. If an abstract was not available, the title was screened for eligibility and the full text was acquired. FKD and TR read the full text in completion to determine their suitability for full text inclusion and data extraction. A discussion resolved differences of opinion between the reviewers. The authors of the selected articles were contacted to provide missing data or to clarify any confusions, if deemed necessary by the authors.

### Data extraction

Data was independently extracted from included studies by two reviewers (FKD and TR). The following data were extracted: (1) Study data: study design, authors, year of publication (2) participants: age, sex, inclusion and exclusion conditions, sample size, (3) results: diagnostic criteria for dental caries at baseline and at the conclusion of the study. When data from articles were missing, the authors were traced. If the authors were deceased, an email was sent to the corresponding or co-authors of the study. The reason for exclusion of articles can be found in the Supplementary section, Additional file 1: Table S1. The list of excluded articles with reasons can be found in Additional file 2: Table S2.

### Critical appraisal

The JBI Critical appraisal tools for cross-sectional studies and cohort studies were employed to determine the quality of the evidence of included articles, Additional file 3: Table S3 and Additional file 4: Table S4.

### Data synthesis

Data were pooled into descriptive tables, and assessed for similarities and differences. Once similar outcomes and comparisons were identified, a meta-analysis was performed and an odds ratio was conducted. If two or more studies were examined in a meta-analysis, a random-effects model was utilized [15]. An I^2^ cut off point to assess heterogeneity was not used [15]. Meta-analysis was performed using STATA 17, and an odds ratio was reported.

### Patient and public involvement statement

Patients or the public were not involved in the design, or conduct, or reporting, or dissemination plans of our research.

## Results

### Search and selection results

This review was conducted between November 2020 and January 2021. The searches on the electronic databases led to the retrieval of 1366 studies. Regular team meetings were held to settle any disputes or differences between the inclusions of the reviewers. After analysing the titles and abstracts, 48 articles were presented for full-text analysis. Following the eligibility criteria, 25 articles were excluded, and 23 were included for the present review.

Papers were uploaded into Rayyan [16] and screened in two stages. The titles and abstracts of suitable studies were independently reviewed by two of our authors against the inclusion criteria, as set out by the published protocol paper [15].

A full text review was performed when a study that met the inclusion criteria was selected or when there was insufficient information based on the title/abstract alone. All disagreements with respect to inclusion of articles were reviewed by the third reviewer for final inclusion. The searching process included all eligible studies until 15th November 2020. Once articles were included, and if any uncertainties were found, the authors of the original articles were contacted for clarification.

### Characteristics of included studies

Table 1 displays detailed information on the characteristics of the studies. Considerable heterogeneity was found in socio-economic status (SES) and education levels. All the studies except four employed a cross-sectional study design [17–20].

**Table 1:** Risk factors and risk indicators evaluated for its association with Early Childhood Caries

Socio-demographic factors	Dietary factors	Oral hygiene	Factors related to breast/bottle feeding	Oral bacterial flora	Other factors
Social class [8, 21–24]	Frequency of sugar intake per day [7, 25–27]	Lack of OH instructions [26]	Sweetened infant beverages [25, 26, 28]	Level of plaque present [7, 29]	Distance to nearest oral health facility [25, 28]
Parents education [21, 22, 25, 28, 30]	Maternal sugar intake frequency [26]	Higher plaque levels [7, 29]	Mode of feeding breast/bottle [25, 26, 28, 31, 32]	Lactobacillus presence [6, 29]	Knowledge of the presence of primary teeth important [30, 32]
SES of parents [21, 23, 25, 26, 33]	Calorie intake [19]	Sources of OHI [26]	Put child to sleep with a bottle [30, 34]	*Streptococcus mutans* presence [6, 27, 29, 35]	Plain water after brushing teeth [30, 32]
Single mothers [24, 30, 34]	Carbohydrate intake [19]	Brushing frequency [25, 26, 28, 30, 32, 36]	Knowledge that frequent and prolonged bottle-feeding can cause dental caries [30]	Salivary flow rate [29]	Parents examine child’s teeth daily [24, 30]
Occupation of caregivers [21, 24, 25, 30, 34]	Fibre intake [19]	Delayed brushing [24, 26]	Length of EBF [37]	Salivary buffering capacity [29]	Parents’ knowledge of sugar in medication [24, 34]
Rural [7, 23, 25, 27–29, 37–39]	Knowledge caries potential sugar [24, 30]	Brushing prevalence [24, 25]	Contents of bottle [24, 34]	Veilonella/Actinomyces /Lactobacillus presence [6]	Fluoride supplementation [34]
	Total sucrose intake [7, 37–40]	Knowledge of oral health [24, 26, 30]	Mechanism of bottle/breastfeeding [25, 26, 28, 31, 32]		Reason for dental visit [24, 30, 34]
	Added sugar [19, 24]	Parental brushing of child’s teeth [25, 28, 30, 32]	Knowledge of sugars in medication [24, 28]		Prenatal supplementation [34]
	Age at the introduction to SSB and sugar-sweetened food [26]	Use toothpaste when brushing [24, 30, 34]	Contents of bottle [24, 28]		Maternal prenatal medical history [34]
	Reasons for giving sugar [26]	Use of toothbrush or cloth [32]			Perinatal complications [34]
	Mode of sugar intake [26, 37]	Children cleaned teeth by themselves [30, 32, 34]			Fluoride levels [23, 27, 41]
	Wasting and stunting [42]	Knowledge of when to start brushing teeth [24, 30]			Age [7, 17, 21, 22, 25, 30–32, 38]
	Micronutrient intake [18, 20, 28]	Parents knowledge of the importance of primary teeth [24, 26, 30]			
	Macronutrient intake [18, 20, 28]	Knowledge of dental plaque [24, 26, 30]			

**Fig. 1:**
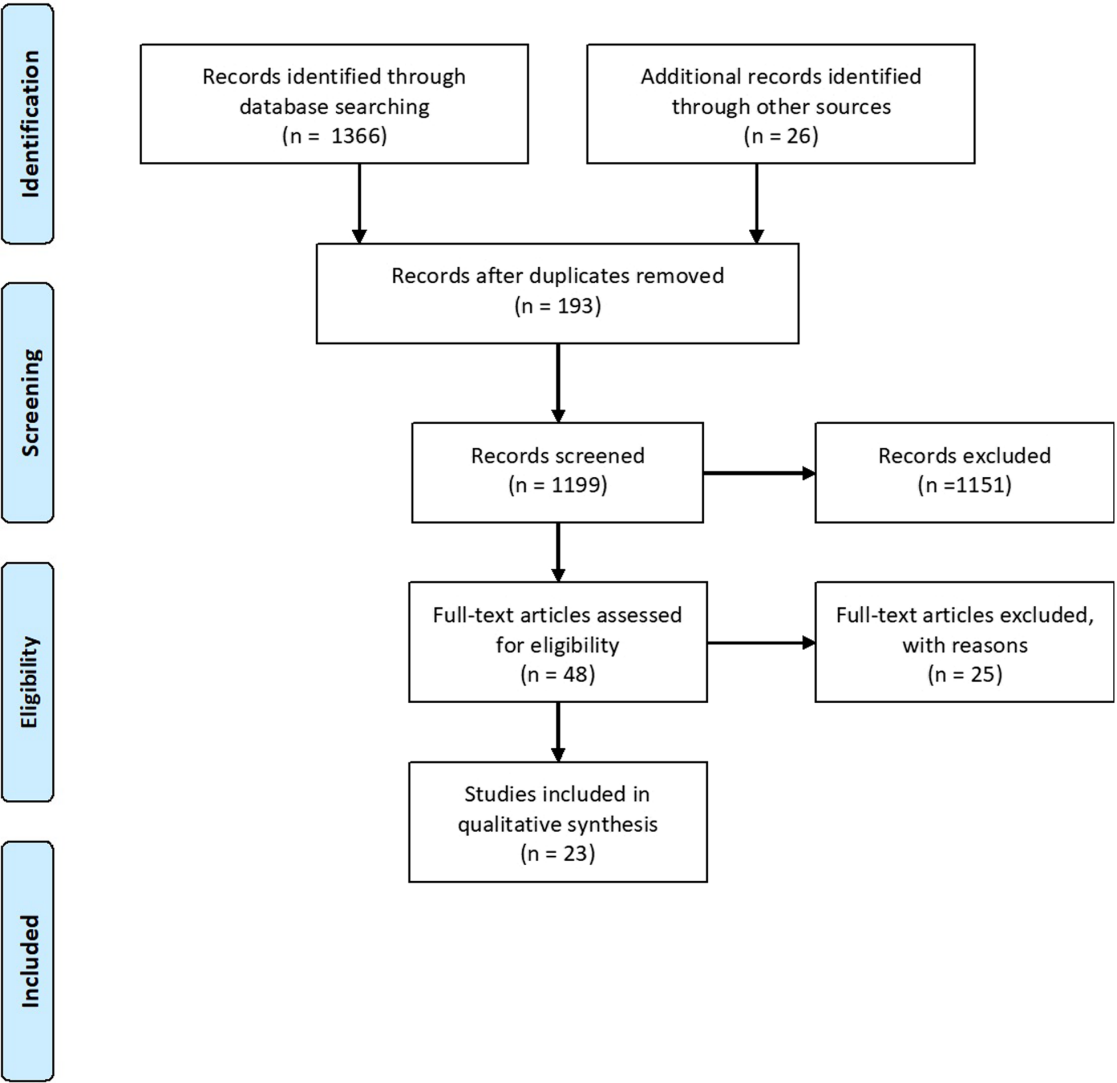
Prisma flow chart of included studies

A total of 58 factors were evaluated for their possible association with the prevalence or incidence of caries found in 23 studies (Fig. 1). These could be grouped into six demographic factors, 14 dietary factors, six factors related to breast and/or bottle-feeding, 14 related to oral hygiene habits, six related to oral bacteria flora and 12 related to other factors such as distance to the nearest oral health facility.

### Risk factors and risk indicators associated with early childhood caries

#### Socio-demographic factors

There was a statistically significant higher caries prevalence in children born to parents from low-income groups than children born to parents of high-income groups [3, 22]. However, Khan et al. [21] found no statistically significant association between socio-economic status and caries prevalence. Parents who were professionals had children with a lower caries prevalence than children of parents who were farmers or unskilled workers [22]. However, Khan et al. [21] found that children of parents of a higher social class had a higher caries prevalence than children of lower social class.

**Fig. 2:**
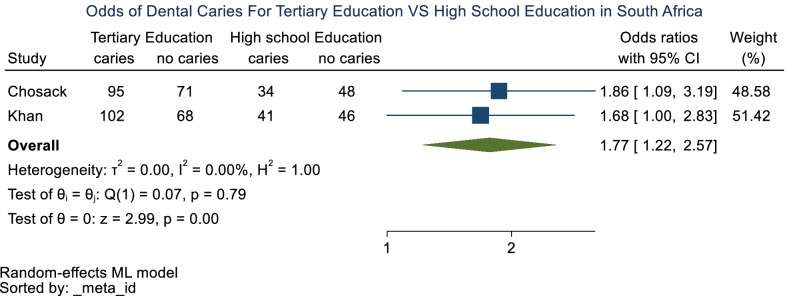
Forest plot of tertiary education versus secondary education and dental caries prevalence

Parents of a lower education group had higher caries prevalence than children of parents with a higher education group [21, 22]. Mohamed and Barnes [34] found that parents of children had jobs that did not require a tertiary education. In addition, Molete and Phakela [25] found that most caregivers had a high school education. The results were supported by Ntombela and Mndzebele [30] who found a caries prevalence of almost 60%, where 90% of the caregivers had a primary school education. Meta-Analysis was performed using random-effects models and concluded that parents of children who had a tertiary education had a 1.77 [1.22–2.57] odds of experiencing dental caries (Fig. 2) compared to those who had a high school education. There was no difference in the odds of children of parents who were from low and high SES (Additional file 10: Figure S1).

Having one or both parents employed had no difference between the caries prevalence of children younger than 71 months [21]. There appears to be conflicting evidence surrounding the prevalence of caries and its’ prevalence to the number of single caregivers. Ntombela and Mndzebele [30] found there to be a more significant number of single caregivers (91%) when the prevalence was low (13.6%), and Mohamed and Barnes (2018) found that the number of single caregivers was 39.5% for a population of children whom all needed dental care [34] There was no difference in caries prevalence between children who came from crowded housing than children who did not come from crowded housing [21]. The absence or presence of electricity in households did not affect children’s caries prevalence [21]. There was no difference in caries prevalence in children whose households had piped water or not. Children from households with electricity had a higher caries prevalence than children who did not have electricity at home. However, this difference was not statistically significant [21]. There was no difference in caries prevalence in children whose households had garbage collection or no garbage collection [21]. Overall, urban children appear to have a higher prevalence, dmft and sugar intake than rural children [7, 23, 25, 27–29, 37–39]. However, rural children had a higher plaque index [29]. The list of sociodemographic factors can be found in Additional file 5: Table S5.

#### Dietary factors

Sugar frequency was reported with variable outcomes. A higher sucrose intake was either associated with a higher caries prevalence or a statistically significant difference in the frequency of sugar intake and caries prevalence [26]. However, we know that a mother’s sugar consumption habits influence her child’s calorie, carbohydrate and fibre intake [26], but were not statistically associated with caries prevalence [19, 24]. Caregiver knowledge of the dangers of sugar consumption and its relationship to caries incidence varied. Gordon [24] found that most caregivers thought that sugar intake and caries were related. However, Ntombela and Mndzebele [30] reported that two-thirds of the caregivers did not know that sugar consumption could increase caries development.

Total sucrose intake was higher in urban than rural populations, and caries prevalence was lower when total sugar intake was lower [7]. Children were introduced to sugars mostly at 6 months and were all ingesting added sugars at 12 months [26]. The majority of the parents reported no specific reason for sugar being added to the children’s diet [26]. Furthermore, the majority of the children consumed their added sugar through bottle feeding [26, 37]. Children who were wasted showed a higher caries prevalence as their wasting severity increased [42]. However, there was a 50% reduction in caries prevalence in children with moderate stunting than those with severe stunting [42].

There was no association between micronutrients (except iron, magnesium, and thiamine) and caries development [18]. The list of dietary factors can be found in Additional file 6: Table S6.

#### Factors related to oral hygiene practices

In the present investigation, some studies collected oral hygiene practices through direct observation using a plaque index or reported behaviours. Table 1 shows the myriad of risk factors or risk indicators for dental caries. Factors such as frequency of toothbrushing [25, 26, 28, 30, 32, 36], plaque visibility [7, 29], age at which toothbrushing started [24, 26], parental supervision of toothbrushing [25, 28, 30, 32], and not having teeth brushed at bedtime. Also, lack of oral hygiene instructions [26] or the source of oral hygiene instructions [26] has a relationship with the prevalence of dental caries. Poor oral health knowledge is negatively associated with ECC [26, 30]. Furthermore, delayed toothbrushing habits (as late as 12–24 months) [24, 26] or using cloth instead of a toothbrush [32], are associated with dental caries. The list of oral hygiene factors can be found in Additional file 7: Table S7.

#### Factors related to breast or bottle-feeding

The mothers of infants as young as 6 months added sugar to infant beverages [26]. A study conducted by Mohamed and Barnes [34] reported that 86% of the study’s population drank cow’s milk, 62% drank fruit juice from a bottle, and 36.9% drank sugar with tea. The authors concluded that breast and bottle feeding [25, 26, 32] were risk indicators for ECC. The majority of mothers’ bottle fed their children [26], and if bottle-fed, 68% added sugar to the bottle. However, Roberts et al. [31] reported no difference in caries between bottle-fed, breastfed or mixed bottle and breastfed infants. Most mothers put their child to sleep with a bottle [30, 34] or fed on demand through the night; both practices are high-risk for ECC. There was no difference in odds of caries prevalence in bottle-only or breast-fed only feeding practices (Additional file 11: Figure S2). There was also no difference in odds of mixed-breast and bottle-fed and bottle-fed only babies (Additional file 12: Figure S3). The list of bottle- and breast-feeding practices can be found in Additional file 8: Table S8.

#### Oral bacterial flora

The level of plaque present was associated with an increase in dmfs score [7, 29]. Lactobacillus presence was associated with an increase in dmfs [29]. However, there was no relationship between dmfs scores and Lactobacillus, Actinomyces or Veillonella numbers [6]. In addition, *Streptococcus Mutans* was associated with an increase in dmfs and dmft score [29, 35]. Salivary flow rate and salivary buffering capacity were not associated with ECC [29]. The list of oral bacterial flora factors can be found in Additional file 9: Table S9.

#### Other factors

A distance further than 5 km to an oral health care [25, 28] was not associated with a lower prevalence of dental disease. Only 8% of caregivers of caries-free children felt that it was essential to have a regular dental check-up to maintain good oral health [30]. The majority of caregivers of children with a low prevalence of dental caries looked into their children’s mouths daily [24, 30].

## Discussion

### Summary of main findings

The importance of this systematic review was to determine the risk factors and risk indicators associated with ECC in SA. Hand-searching the included articles to identify additional references and papers relevant to this SR increased the study’s validity. However, unpublished theses were excluded, which could have contained more risk-causing data, which is a limitation of this study.

Longitudinal studies are rare and expensive, we cannot expect too many risk factors associated with ECC, but rather risk indicators. There are four longitudinal studies in the present study. More weight should be allocated to longitudinal studies instead of cross-sectional or case–control studies, as the former benefit from actual causation rather than mere associations.

This review evaluated studies that determined predictors of dental caries but were cross-sectional in design, which was a huge flaw as predictors need to be analysed using longitudinal studies. In addition, there is considerable reliance on historical data, which is subjected to recall bias, especially breastfeeding practices if the child has already been weaned off the breast.

While global evidence has revealed risk factors socio-economic factors, dietary factors and oral hygiene factors for ECC in children [5], South African children face additional factors due to the lingering effects of Apartheid such as a lack of access to quality oral healthcare oral health education and food. Access to food is a problem in South Africa, where 17.3% of the population is subjected to food insecurity [43]. In addition, there are policies that are directed towards Early Childhood Development [44, 45] which does not include any policies towards ECC prevention or its management.

### Agreements and disagreements with previous studies

It is interesting to note that Mutans Streptococcus (MS) concentrations were only associated with dmft and dmfs score but not associated with caries’ prevalence [6, 27, 29, 35]. Parisotto et al. [46] and Kirthiga et al. [5] found MS to be a significant ECC predictor. It is also noteworthy that children of higher-income parents participating in the study had a higher caries experience. However, they may also have had more access to sweets [38]. Khan and Cleaton-Jones [21] reported [47] similar findings in infants whose parents had a higher education than those whose parents had middle-level education, which was the only significant finding the authors reported from a substantial sample of infants. However, Burt and Pai [47] felt that with fluoridated toothpaste or sugar should not play a role in ECC development.

Other non-significant findings were social class, caregivers' employment status, education level, home crowding, access to piped water, and garbage collection presence. We could speculate that income, education, sugar and caries are all positively correlated with each other.

A third of the total population in South Africa is rural [48]. Nevertheless, there is no consensus about whether being part of a rural community increases or decreases the risk of developing ECC. Even though Cleaton-Jones et al. [7] found a higher caries prevalence in rural children than urban children, this difference was not statistically significant. Granath et al. [29] found that rural children had a higher plaque index, a higher gingival index, a higher salivary flow and salivary buffering capacity but a lower lactobacillus, lower SM concentrations with lower dmfs scores compared to urban children. There is a higher education level in urban areas, explaining the lower caries prevalence rates in the urban areas [21, 22].

Many studies were conducted during the Apartheid era and reported the ethnicity of the infants. Evidence reporting the similarities between ethnic groups far outweigh the differences, and the most potent mediator was poverty [49]^.^ Thus, the reviewers chose not to report ethnicity and collapsed the data by age or urban/rural locality instead. Nutrition influences teeth during the pre-eruptive stages, including prenatal, perinatal and postnatal developments. Diet and nutrition have an important impact on ECC development [50].

Policymakers and health managers can use this study to customise prevention and intervention programs for a South African population, in which the majority of the population are dependent on the state for preventative and curative services.

## Conclusions

More high-quality cohort studies need to be performed to evaluate true risk factors for ECC in a South African setting. In addition, parental/caregiver oral educational knowledge needs promoting before the emergence of their children’s teeth. Previous systematic reviews only considered *Streptococcus Mutans*, sucrose intake and breast and bottle-feeding practices. However, the social determinants of health were excluded. In South Africa, due to its high inequality and level of poverty, the social determinants of health needs to be incorporated in future studies, and suitable targeted interventions need to be developed and implemented. This review can add to existing policies aimed at early childhood development as current policies lack any recommendations or strategies aimed at preventing ECC.

## Supplementary Information

**Additional file 1**.** Supplementary Table 1**. Exclusion criteria applied.

**Additional file 2**.** Supplementary Table 2**. List of excluded articles.

**Additional file 3**.** Supplementary Table 3**. Critical appraisal of cross-sectional studies.

**Additional file 4**.** Supplementary Table 4**. Critical Appraisal of Cohort Studies.

**Additional file 5**.** Supplementary Table 5**. Sociodemographic factors associated with ECC in South Africa.

**Additional file 6**.** Supplementary Table 6**. Socio demographic factors

**Additional file 7**.** Supplementary file Table 7**. Oral hygiene factors.

**Additional file 8**.** Supplementary file Table 8**. Factors related to bottle or breastfeeding.

**Additional file 9**.** Supplementary file Table 9**. Oral bacterial flora.

**Additional file 10**.** Supplementary file-Figure 1**. Forest Plot of low SES VS high SES and dental caries.

**Additional file 11**.** Supplementary file-Figure 2**. Forest plot of bottle-fed only VS breastfed only practices and dental caries prevalence.

**Additional file 12**.** Supplementary file-Figure 3**. Forest plot of breast- and bottle-fed VS bottle- fed only practices and dental caries prevalence.

## Data Availability

Dataset available from the kikapu repository, https://doi.org/10.25379/uwc.14679273.v1. Accessed 17/01/2022.

## Abbreviations

dmft: Decayed missing and filled teeth
dmfs: Decayed missing and filled surface
MS: Mutans Streptococci
UWC: University of the Western Cape
n/a: Not applicable
