# Supplementary material for: Maternal and infant risk factors and risk indicators associated with early childhood caries in South Africa: a systematic review
Source: BMC Oral Health. 2022 May 18;22:183. doi: 10.1186/s12903-022-02218-x (PMC9118582; doi:10.1186/s12903-022-02218-x)
Supplement: Supplementary file 1 — Additional file 1. Supplementary Table 1. Exclusion criteria applied. [file 12903_2022_2218_MOESM1_ESM.pdf]

Supplementary file- Table 1: Exclusion criteria applied

- Animal, ecological, and in vitro studies; human studies on experimental caries; case reports
- Reviews, letters and editorials
- Studies with no caries experience
- Studies in children 6 years and older
