# Supplementary material for: Maternal and infant risk factors and risk indicators associated with early childhood caries in South Africa: a systematic review
Source: BMC Oral Health. 2022 May 18;22:183. doi: 10.1186/s12903-022-02218-x (PMC9118582; doi:10.1186/s12903-022-02218-x)
Supplement: Supplementary file 2 — Additional file 2. Supplementary Table 2. List of excluded articles. [file 12903_2022_2218_MOESM2_ESM.pdf]

Supplementary Table 2: List of excluded articles

Article	Reason for exclusion
MacIntyre, U.E. and Du Plessis, J.B., 2006. Dietary intakes and caries experience in children in Limpopo Province, South Africa. SADJ: journal of the South African Dental Association= tydskrif van die Suid-Afrikaanse Tandheelkundige Vereniging, 61(2), pp.58-63.	Wrong population
Sullivan, Å., Granath, L. and Widenheim, J., 1989. Correlation between child caries incidence and S. mutans/lactobacilli in saliva after correction for confounding factors. Community dentistry and oral epidemiology, 17(5), pp.240-244.	Wrong population
Thekiso, M., Yengopal, V., Rudolph, M.J. and Bhayat, A., 2012. Caries status among children in the West Rand District of Gauteng Province, South Africa. SADJ: journal of the South African Dental Association= tydskrif van die Suid-Afrikaanse Tandheelkundige Vereniging, 67(7), pp.318-320.	No Risk factors
Matejka, J., Sinwel, R., Cleaton-Jones, P., Williams, S., Hargreaves, J.A., Fatti, L.P. and Docrat, M., 1989. Dental caries at five and twelve years in a South African Indian community: a longitudinal study. International journal of epidemiology, 18(2), pp.423-426.	No Risk factors
du Plessis, J.B., 2000. The oral health status in Mahonisi: a community with very low levels of dental caries. SADJ: journal of the South African Dental Association= tydskrif van die Suid-Afrikaanse Tandheelkundige Vereniging, 55(6), pp.308-312.	No Risk factors
Moola, R.J., 1988. Prevalence of dental caries in preschool and primary school children in Mamre. South African Medical Journal, 74(7), pp.344-346.	No Risk factors
MacKeown, J.M., Cleaton-Jones, P.E. and Perdraut, G.G., 1996. Energy and macronutrient intake of one-year-old South African urban children: The Birth to Ten (BTT) Study. Paediatric and Perinatal Epidemiology, 10(2), pp.150-160.	No dmft scores
Richardson, B.D., Rantsho, J.M. and Pieters, L., 1978. Total sucrose intake and dental caries in Black and White South African children of 1-6 years. Part I: Sucrose intake.	No dmft scores
MacKeown, J.M. and Faber, M., 2002. Urbanisation and cariogenic food habits among 4–24-month-old black South African children in rural and urban areas. Public health nutrition, 5(6), pp.719-726.	No dmft scores
Walker, A.R.P. and Cleaton-Jones, P.E., 1975. Dental caries and sugar intake. The Lancet, 306(7938), p.765.	Letter
Molete, M., 2018. Children's oral health in South Africa: Time for action. South African Journal of Child Health, 12(4), pp.133-133.	Letter
Naidoo, S. and Myburgh, N., 2007. Nutrition, oral health and the young child. Maternal & child nutrition, 3(4), pp.312-321.	Review
Marshall, T.A., 2014. Low intake of sugars may reduce risk of dental caries. Journal of Evidence Based Dental Practice, 14(2), pp.56-58.	Review

Gordon N. Oral health care for children attending a malnutrition clinic in South Africa. Int J Dent Hyg. 2007 Aug;5(3):180-6. doi: 10.1111/j.1601-5037.2007.00261.x. PMID: 17615028.	Wrong population
Cleaton-Jones P, Williams S, Fatti P. Surveillance of primary dentition caries in Germiston, South Africa, 1981-97. Community Dent Oral Epidemiol. 2000 Aug;28(4):267-73. doi: 10.1034/j.1600-0528.2000.280404.x. PMID: 10901405.	Wrong outcome
Mohamed N, Barnes JM. Early childhood caries and dental treatment need in low socio-economic communities in Cape Town, South Africa. Health SA. 2018 Jul 12;23:1039. doi: 10.4102/hsag.v23i0.1039. PMID: 31934368; PMCID: PMC6917374.	No Risk factors
Marx J, Pretorius E. Asthma--a risk factor for dental caries. SADJ. 2004 Sep;59(8):323, 325-6. PMID: 15559914.	Review
**Caries risk prediction--the way of the future.**	Review
Li Y, Navia JM, Caufield PW. Colonization by mutans streptococci in the mouths of 3- and 4-year-old Chinese children with or without enamel hypoplasia. Arch Oral Biol. 1994 Dec;39(12):1057-62. doi: 10.1016/0003-9969(94)90058-2. PMID: 7717887.	Wrong population
Lalloo R, Myburgh NG, Hobdell MH. Dental caries, socio-economic development and national oral health policies. Int Dent J. 1999 Aug;49(4):196-202. doi: 10.1111/j.1875-595x.1999.tb00522.x. PMID: 10858754.	Review
Nqcobo CB, Yengopal V, Rudolph MJ, Thekiso M, Joosab Z. Dental caries prevalence in children attending special needs schools in Johannesburg, Gauteng Province, South Africa. SADJ. 2012 Aug;67(7):308-13. PMID: 23951782.	Wrong population
Virtanen JI, Bloigu RS, Larmas MA. Effect of early or late eruption of permanent teeth on caries susceptibility. J Dent. 1996 Jul;24(4):245-50. doi: 10.1016/0300-5712(95)00078-x. PMID: 8783528.	Wrong population
Linke HA, Kuyinu EO, Ogundare B, Imam MM, Khan SH, Olawoye OO, LeGeros RZ. Microbiological composition of whole saliva and caries experience in minority populations. Dent Clin North Am. 2003 Jan;47(1):67-85, ix. doi: 10.1016/s0011-8532(02)00060-5. PMID: 12519006.	Wrong population
Coogan MM, Mackeown JM, Galpin JS, Fatti LP. Microbiological impressions of teeth, saliva and dietary fibre can predict caries activity. J Dent. 2008 Nov;36(11):892-9. doi: 10.1016/j.jdent.2008.07.004. Epub 2008 Aug 28. PMID: 18760520.	Wrong population
Walker AR. Nutritional and dental implications of high and low intakes of sugar. Int J Food Sci Nutr. 1995 May;46(2):161-9. doi: 10.3109/09637489509012545. PMID: 7621089.	Review
