# Supplementary material for: Maternal and infant risk factors and risk indicators associated with early childhood caries in South Africa: a systematic review
Source: BMC Oral Health. 2022 May 18;22:183. doi: 10.1186/s12903-022-02218-x (PMC9118582; doi:10.1186/s12903-022-02218-x)
Supplement: Supplementary file 3 — Additional file 3. Supplementary Table 3. Critical appraisal of cross-sectional studies. [file 12903_2022_2218_MOESM3_ESM.pdf]

Supplementary Table 3: Critical appraisal of cross-sectional studies

Author	year	Were the criteria for inclusion in the sample clearly defined?	Were the study subjects and the setting described in detail?	Was the exposure measured in a valid and reliable way?	Were objective, standard criteria used for measurement of the condition?	Were confounding factors identified?	Were strategies to deal with confounding factors stated?	Were the outcomes measured in a valid and reliable way?	Was appropriate statistical analysis used?	Score
Chosack	1988	yes	no	yes	yes	yes	yes	yes	unsure	6
Chosack	1990	yes	yes	yes	yes	yes	no	yes	yes	7
Cleaton Jones	2000	yes	yes	yes	yes	yes	yes	no	none	6
Cleaton Jones	1984	yes	yes	yes	yes	yes	no	yes	none performed	6
Gordon	1985	yes	yes	yes	yes	yes	no	yes	none	6
Gordon, N	2007	yes	no	no	no	yes	no	no	none	2
Granath	1991	yes	yes	yes	yes	no	no	yes, but not reported	none performed	4
Granath	1993	yes	yes	yes	yes	yes	no	yes	no	6
Khan	1998	yes	yes	yes	yes	yes	yes	yes, but not reported	yes	7
Mndzebele	2014	yes	yes	yes	yes	yes	yes	yes	yes	8
Mohamed	2018	yes	yes	none	yes	no	n/a	unsure	no	3
Molete	2018	yes	yes	yes	yes	yes	no	yes	no	6
Ntombela	2015	yes	yes	yes	yes	no	none	no	yes	5
Postma	2008	yes	yes	yes	yes	yes	yes	yes	yes	8
Richardson	1981	no	no	yes	yes	no	no	yes	none	3
Richardson	1979	yes	yes	not sure	yes	no	no	no	no	3
Richardson	1978	no	yes	yes	no	yes	yes	no	none	4
Roberts	1993	yes	unsure	no	yes	yes	yes	yes	yes	6
Toi	1998	yes	yes	yes	yes	no	n/a	no	no	4
