# Supplementary material for: Maternal and infant risk factors and risk indicators associated with early childhood caries in South Africa: a systematic review
Source: BMC Oral Health. 2022 May 18;22:183. doi: 10.1186/s12903-022-02218-x (PMC9118582; doi:10.1186/s12903-022-02218-x)
Supplement: Supplementary file 4 — Additional file 4. Supplementary Table 4. Critical Appraisal of Cohort Studies. [file 12903_2022_2218_MOESM4_ESM.pdf]

Supplementary Table 4: Critical Appraisal of Cohort Studies

Author	Year of publication	Study Design	Were the two groups similar and recruited from the same population?	Were the exposures measured similarly to assign people to both exposed and unexposed groups?	Was the exposure measured in a valid and reliable way?	Were confounding factors identified?	Were strategies to deal with confounding factors stated?	Were the groups/participants free of the outcome at the start of the study (or at the moment of exposure)?	Were the outcomes measured in a valid and reliable way?	Was the follow up time reported and sufficient to be long enough for outcomes to occur?	Was follow up complete, and if not, were the reasons to loss to follow up described and explored?	Were strategies to address incomplete follow up utilized?	Was appropriate statistical analysis used?	Score
Mackeown	2003	cohort	NA	NA	yes	yes	no	yes	yes	yes	yes	no	no	6
Mackeown	2001	cohort	NA	NA	NA	no	no	yes	yes	yes	no	no	yes	4
Mackeown	2000	cohort	NA	NA	yes	no	no	yes	yes	yes	yes	no	no	5
Williams	1985	cohort	NA	NA	yes	no	no	no	yes	yes	NA	no	no	3
