# Supplementary material for: Maternal and infant risk factors and risk indicators associated with early childhood caries in South Africa: a systematic review
Source: BMC Oral Health. 2022 May 18;22:183. doi: 10.1186/s12903-022-02218-x (PMC9118582; doi:10.1186/s12903-022-02218-x)
Supplement: Supplementary file 5 — Additional file 5. Supplementary Table 5. Sociodemographic factors associated with ECC in South Africa. [file 12903_2022_2218_MOESM5_ESM.pdf]

Supplementary Table 5: Socio-Demographic Factors

Socio demographic factors											
Article Number	Article		year	Study design	Age	Social Class	Parents education	SES of parents	Single Caregivers	Occupation of caregivers	Rural
1	Caries prevalence and severity in the primary dentition and Streptococcus mutans levels in the saliva of preschoolchildren in South Africa.	Chosack	1988	cross-sectional	3-5						
2	Social class, parent's education and dental caries in 3 to 5 year old children	Chosack	1990	cross-sectional	3-5	increase in dmft;increase dmfs; higher prevalence in lower Social class. The caries prevalence of babies of parents in high income (IIIM, IV, V) to low income (I,II,IIIN) parents is OR 0.03 [0.00 to 0.20]./	higher prevalence if tech college or university; higher dmft score and higher dmfs score if secondary school, OR=1.86				
3	Nutritional status and dental caries in a large sample if 4- and 5- year olds south african children	Cleaton Jones	2000	cross-sectional	4-5						
4	Dental caries and sucrose intake in five south african preschool groups	Cleaton Jones	1984	cross-sectional	2-5						higher caries prevalence compared to urban children (2 y.o. (SS) and 4 y.o. (NSS)); higher dmft in 2 y.o. rural kids (SS); rural black lower mean dmft compared to urban kids (5 y.o.) (SS)

5	Prevalence of dental caries, patterns of sugar consumption and oral hygiene practices in infancy in S. Africa.	Gordon	1985	cross-sectional	1-2			more semi skilled and unskilled parents in the population			
6	Oral health care for children attending a malnutrition clinic in SA	Gordon, N	2007	cross-sectional	1-4	86% lived in brick houses			55% were single mothers	38% were employed	
7	Correlations between caries prevalence and potential etiologic factors in large samples of 4-5-yr-old children.	Granath	1991	cross-sectional	4-5						Rural children had: a higher plaque index; higher Gingival bleeding; higher salivary glow; higher salvary buffering capacity but lower lactbacillus culture and lower SM concentrations and lower dmfs levels
8	Prevalence of dental caries in 4- to 5-year-old children partly explained by presence of salivary mutans streptococci	Granath	1993	cross-sectional	4-5						dmfs score was higher in urban [5.9-12.4] than rural [3.6] children.
9	Dental caries in African preschool children: Social factors as disease markers	Khan	1998	cross-sectional	3-5	No association. 1.42 [0.89 to 2.27]	higher education showed a higher odds of caries prevalence compared to middle level education, p < 0.05. 1.68 [1.00 to 2.83]	no association between various income groups; no association between home crowded or not; no association between piped water absence or presence; no association ebtween garbage collection or not		no association between unemployment of both parents, 1.03 [0.52 to 2.04]. both unemployed to employment of only one parent,1.04 [0.54 to 2.02];	
10	Caries and micronutrient intake among urban South African children: a cohort study.	Mackeown	2003	cohort	5						
11	Dental caries incidence in relation to nutrient intake in urban preschool children	Mackeown	2001	cohort	5						

12	Energy and macronutrient intake in relation to dental caries incidence in urban black south African preschool children in 1991 and 1995: the Birth to ten study	Mackeown	2000	cohort	1-5						
13	Prevalence and causes of ECC in children less than 6 years old at Tembisa Hospital, SA	Mndzebele	2014	cross-sectional	<6						
14	Characteristics of Children Under 6 Years of Age Treated for Early Chidhood Caries in South Africa.	Mohamed	2018	cross-sectional	<6		Very few parents had jobs that required a tertiary qualifica-tion. M		39.2% (n=55). N=140	n=86,45.5% unemployed; 36% employed; 33% unavailable data. Most occupations listed were those of blue-collar work- ers including jobs such as drivers, general workers and builders among the fathers. Four of the fathers were self-employed. The majority of the employed mothers listed jobs such as assistants, packers and cleaners.	Urban
15	ECC experience of children accessing selected immunisation facilities in JHB	Molete	2018	cross-sectional	6mo-6 years		n=321 had a high school education; 84 had a tertiary education and 40 had a primary school education	majority parents were unemployed (n=310,69.5%)		310 (69.5 %) were unemployed; 131 were employed and 5 were still at school	Urban
16	Parents influence on Early childhood caries among their children at a community health centre in Gauteng Province	Ntombela	2015	cross-sectional	1-5		91% had a primary education		73% were single; 27% were married	63% were unemployed and 37 % were employed	
17	Socio-demographic correlates of early childhood caries	Postma	2008	cross-sectional	36-71mo	Children of high income, 8.29(3.1), 7.77 (3.09) had more sugar expenditure than children					Children in urban,8.06 (2.78), had higher sugar expenditure

	prevalence and severity in a developing country--South Africa.					of umeployed or middle income, 7.63 (1.75). children of middle income parents, had the highest dmft score, 3.10 (3.91) compared to children of high income parents, 2.37 (3.38) and children of Unemployed parents, 2.56 (3.65). children of midddle income parents had the highest caries prevalence, 1533/2610 (58.57%) , followed by children of high income parents, 603/1179 (51.17%), followed by children of parents who were unemployed, 764/1511(50.57%) and				compared to non-urban , 7.35 (1.94) children. DMFT was higher in urban , 3.07 (3.88) cpmpared to non-urban , 2.39 (3.46). caries prevalence was higher on urban, 1891/2528 (59.63%) compared to non-urban , 1550/3171 (48.87%)
18	Sweets, snacks, and dental caries: South African interracial patterns.	Richardson	1981	cross-sectional	3-5					Urban (64+/-15.9)children consuming more sugar than rural children (54+/- 33.6)
19	The bearing of dietary sucrose on the deciduous dentition of pre-school children in Transvaal	Richardson	1979	cross-sectional	1-6					higher total sucrose intake per day in urban (686, 53.1338 (35.1)) compared toi rural (427, 38.4 (31.5)).
20	Total sucrose intake and dental caries in Black and in White South AFrican Children of 1-6 years: Part II	Richardson	1978	cross-sectional	1-6			Children in average SE groups had a lower total sugar intake, n=581, 76.07 (39.05) compareed to the lower SE group, n=557, 60.28 (43.5).		Urban children comsumed more sugar compared to rural children
21	Patterns of breast and bottle feeding and their association with dental caries in 1- to 4-year-old South African children.1. dental caries prevalence and experience. Community Dent Health 1993 10: 405-413	Roberts GJ, Cleaton-Jones PE, Fatti LP et al. Patterns of breast and bottle feeding and their association with dental caries in 1- to 4-year-old South African children. Community Dent	1993	cross-sectional	1-4	The odds of caries prevalence og babies in high (IIIm,IV, V) income to low income parents is (I,II,IIIN) 0.92 [0.72 to 1.19]				

		Health 1993 10: 405-413									
22	Mutans Streptococci and other caries-associated acidogenic bacteria in five-year old children in SA	Toi	1998	cross-sectional	5						
23	Dental caries and dental treatment in the primary dentition in an industrialized South African community.	Williams	1985	cohort	2-5						
