# Supplementary material for: Maternal and infant risk factors and risk indicators associated with early childhood caries in South Africa: a systematic review
Source: BMC Oral Health. 2022 May 18;22:183. doi: 10.1186/s12903-022-02218-x (PMC9118582; doi:10.1186/s12903-022-02218-x)
Supplement: Supplementary file 8 — Additional file 8. Supplementary file Table 8. Factors related to bottle or breastfeeding. [file 12903_2022_2218_MOESM8_ESM.pdf]

Supplementary file: Table 8 Factors related to bottle or breastfeeding

Factors related to bottle or breastfeeding											
Article Number	Article		year	Study design	Sweetened infant beverages	Knowledge that frequent and prolonged bottle feeding can cause dental caries	Put child to sleep with bottle	length of exclusive breastfeeding	Mechanism of bottle feeding breast /bottle	knowledge of sugars in medication	contents of bottle
1	Caries prevalence and severity in the primary dentition and Streptococcus mutans levels in the saliva of preschoolchildren in South Africa.	Chosack	1988	cross-sectional							
2	Social class, parent's education and dental caries in 3 to 5 year old children	Chosack	1990	cross-sectional							
3	Nutritional status and dental caries in a large sample if 4- and 5- year olds south african children	Cleaton Jones	2000	cross-sectional							
4	Dental caries and sucrose intake in five south african preschool groups	Cleaton Jones	1984	cross-sectional							
5	Prevalence of dental caries, patterns of sugar consumption and oral hygiene practices in infancy in S. Africa.	Gordon	1985	cross-sectional	sweetened infant bottle, 53% of 12-24 mo olds				Only 2% of infants were breastfed, while 68% were bottle fed and 30%were both bottle and breastfed. . if bottle fed, 58% parents filled bottle with sugar . of bottle and breastfed, 26% parents added sugar to the bottle.		
6	Oral health care for children attending a malnutrition clinic in SA	Gordon, N	2007	cross-sectional						parents had knowledge that sugars in medication causes caries	For those who were bottle fed, the contents were milk and/or tea, juice and water
7	Correlations between caries prevalence and potential etiologic factors in large samples of 4-5-yr-old children.	Granath	1991	cross-sectional							
8	Prevalence of dental caries in 4- to 5-year-old children partly explained by presence of salivary mutans streptococci	Granath	1993	cross-sectional							
9	Dental caries in African preschool children: Social factors as disease markers	Khan	1998	cross-sectional							
10	Caries and micronutrient intake among urban South African children: a cohort study.	Mackeown	2003	cohort							
11	Dental caries incidence in relation to nutrient intake in urban preschool children	Mackeown	2001	cohort							
12	Energy and macronutrient intake in relation to dental caries incidence in urban black south African preschool children in 1991 and 1995: the Birth to ten study	Mackeown	2000	cohort							
13	Prevalence and causes of ECC in children less than 6 years old at Tembisa Hospital, SA	Mndzebele	2014	cross-sectional					OR=2.32 for bottle fed compared to breast fed in caries prevalence, p < 0,.05; OR=1.58 both breast and bottle comnpared to breastfed alone, p=0.213.		

14	Characteristics of Children Under 6 Years of Age Treated for Early Chidhood Caries in South Africa.	Mohamed	2018	cross-sectional	86% drank cows milk. only 4% of them added sugar/nesquick to the milk. 62% drank fruit juice from the bottle. 8.5% SSB, 36.9% drank tea with sugar (rooibos (32.1%) or ceylon (68.7%).0 had breast milk in bottle; only n=15 recieved formula milk;		101 (93.6%) went to sleep while brest or bottle feeding. 90% (n=126) were fed on demand through the night		n=11, 7.85%, were exclusively breastfed. Breastfeeding stopped at 9 monhts. Bottle feeding stoppped at 2.3 years (28 months(. 18.	Not asked if medication had sugar. But n=20, 14.3%, received medication	The majority of children (86%) drank cow's milk out of the bottle. Of these, 96% of the parents claimed to have added nothing to the milk. A sweetened flavoring (Nesquick®) was added to the bottles of 10.8% of the chil-dren while 8.1% had sugar added to the bottle. Honey was added in only one instance. Of the 129 children in this study, 62% drank fruit juice from the baby bottle. Cola drinks or other soft drinks 8.5% • Tea with sugar 36.9%. Of this group, 31.2 % favored an indigenous herbal tea (rooibos tea) and the rest (68.7%) drank Ceylon tea. • Sugared water 3.8%
15	ECC experience of children accessing selected immunisation facilities in JHB	Molete	2018	cross-sectional	280 (62.78%) placed sugar in tea or porridge				323(72.42%) breastfed; 173 (43.14%) bottle fed		
16	Parents influence on Early childhood caries among their children at a community health centre in Gauteng Province	Ntombela	2015	cross-sectional		and that frequent and prolonged bottle-feeding can cause dental caries (6% vs 1%, p<0.05)	Fifty-two per cent of the respondents had never put their child to bed with a bottle.				
17	Socio-demographic correlates of early childhood caries prevalence and severity in a developing country--South Africa.	Postma	2008	cross-sectional							
18	Sweets, snacks, and dental caries: South African interracial patterns.	Richardson	1981	cross-sectional							
19	The bearing of dietary sucrose on the deciduous dentition of pre-school children in Transvaal	Richardson	1979	cross-sectional				72.5 % breastfed for longer than 6 months. approximately 49-50 weeks long			
20	Total sucrose intake and dental caries in Black and in White South AFrican Children of 1-6 years: Part II	Richardson	1978	cross-sectional							
21	Patterns of breast and bottle feeding and their association with dental caries in 1- to 4-year-old South African children.1. dental caries prevalence and experience. Community Dent Health 1993 10: 405-413	Roberts GJ, Cleaton-Jones PE, Fatti LP et al. Patterns of breast and bottle feeding and their association with dental caries in 1- to 4-year-old South African children. Community Dent Health 1993 10: 405-413	1993	cross-sectional					The odds of caries in bottle and breastfed babies to breastfed babies is 1.15 [0.89 to 1.47]. The odds of caries in bottle fed babies to breastfed babies is 1.02 [0.72 to 1.45]. The odds of caries in Bottle fed to bottle and breastfed babies is 0.89 [0.63 to 1.26].		
22	Mutans Streptococci and other caries-associated acidogenic bacteria in five-year old children in SA	Toi	1998	cross-sectional							
23	Dental caries and dental treatment in the primary dentition in an industrialized South African community.	Williams	1985	cohort							
