# Supplementary figures and images for: Maternal and infant risk factors and risk indicators associated with early childhood caries in South Africa: a systematic review
Source: BMC Oral Health. 2022 May 18;22:183. doi: 10.1186/s12903-022-02218-x (PMC9118582; doi:10.1186/s12903-022-02218-x)

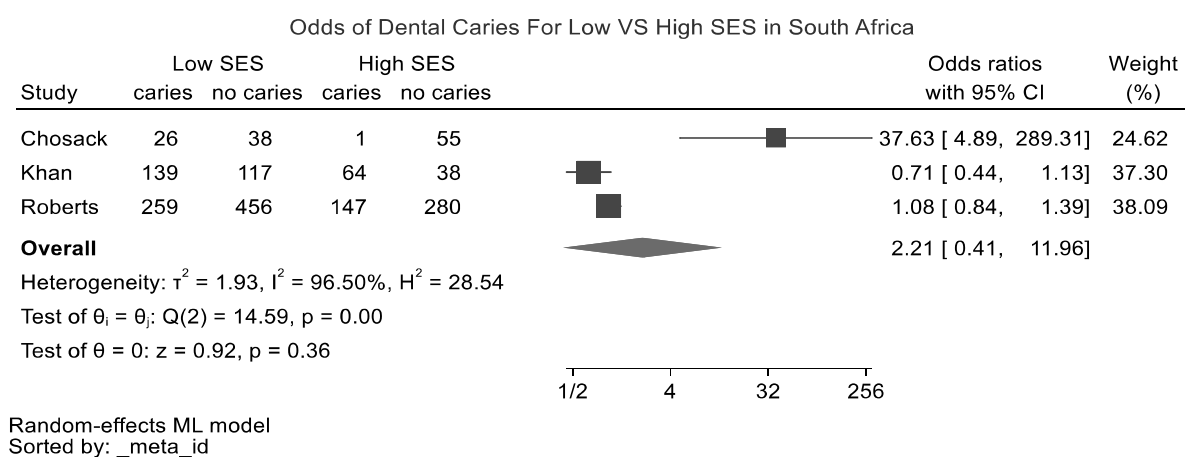

Supplementary file- Figure 1: Forest Plot of low SES VS high SES and dental caries

Supplement: Supplementary file 10 — Additional file 10. Supplementary file-Figure 1. Forest Plot of low SES VS high SES and dental caries. [file 12903_2022_2218_MOESM10_ESM.pdf]
