# Supplementary material for: Maternal and infant risk factors and risk indicators associated with early childhood caries in South Africa: a systematic review
Source: BMC Oral Health. 2022 May 18;22:183. doi: 10.1186/s12903-022-02218-x (PMC9118582; doi:10.1186/s12903-022-02218-x)
Supplement: Supplementary file 11 — Additional file 11. Supplementary file-Figure 2. Forest plot of bottle-fed only VS breastfed only practices and dental caries prevalence. [file 12903_2022_2218_MOESM11_ESM.pdf]

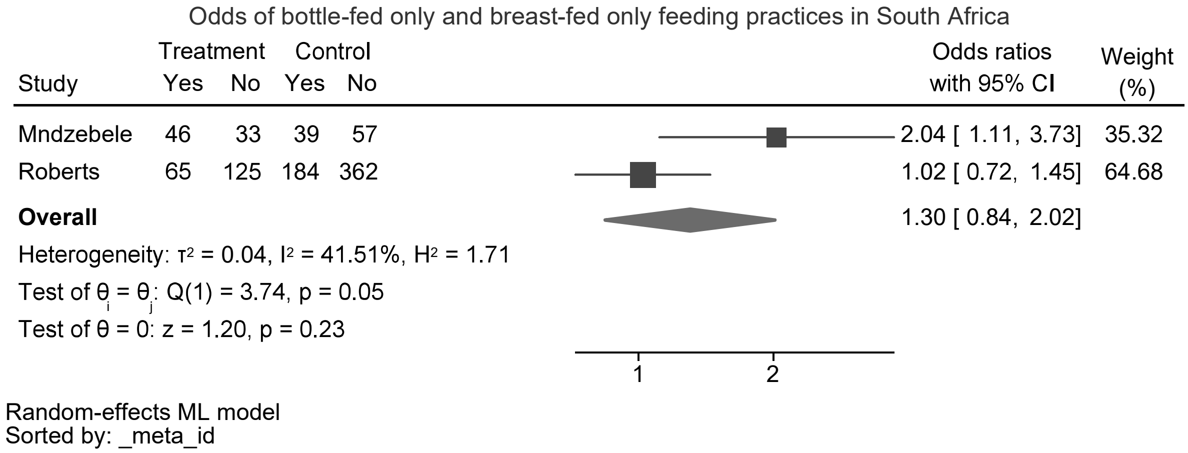

Supplementary file- Figure 2: Forest plot of bottle-fed only VS breast fed only practices and dental caries prevalence
